# Effect of *Cordyceps militaris* Extract on Visceral Adipose Tissue Changes After Ovariectomy in Rodents

**DOI:** 10.1155/ije/6699051

**Published:** 2026-01-29

**Authors:** Kazuya Kusama, Chisaki Fujii, Kanoko Yoshida, Mikihiro Yoshie, Yoshiyuki Adachi, Hiroaki Miyaoka, Kazuhiro Tamura

**Affiliations:** ^1^ Laboratory of Endocrine Pharmacology, Tokyo University of Pharmacy and Life Sciences, Tokyo, 192-0392, Japan, toyaku.ac.jp; ^2^ Laboratory for Immunopharmacology of Microbial Products, Tokyo University of Pharmacy and Life Sciences, Tokyo, 192-0392, Japan, toyaku.ac.jp; ^3^ Department of Biomolecular Organic Chemistry, Tokyo University of Pharmacy and Life Sciences, Tokyo, 192-0392, Japan, toyaku.ac.jp

**Keywords:** adipose differentiation, cellular senescence, *Cordyceps militaris*, ovariectomy

## Abstract

The increasing global prevalence of obesity, exacerbated by factors such as high‐fat diets and reduced physical activity, poses a substantial risk for lifestyle‐related diseases, particularly in postmenopausal women. Premenopausal women initially have a lower incidence of cardiovascular disease than men, but this risk increases after menopause, highlighting menopause as a critical risk factor. Our previous study showed that the extract of *Cordyceps militaris* (CM) modulates androgen metabolism partially by inhibiting the gene expression of catabolizing enzyme 5α‐reductase. In this study, we investigated the effect of CM on estrogen deficiency‐induced obesity in ovariectomized (OVX) mice fed a 0.1% CM diet (estimated human equivalent dose: 7.5 g/day) for 52 days. OVX mice had increased body weight, which subsequently decreased with CM, without altering daily food intake. Regarding visceral fat, CM suppressed OVX‐induced adipogenic markers (*Pparg*, *Cebpa*, *Cebpb*, *Fabp4,* and *Adipoq)* and protein levels of C/EBPβ, PPARγ, and p‐AKT. CM effectively reversed the OVX‐induced reduction in the levels of adipose *Cyp17a1* and *Hsd17b1*, key enzymes involved in steroid hormone biosynthesis, increased the cellular senescence markers *p21* and *p53,* and decreased *Lmnb1* expression. CM reduced the expression of oxidative stress markers HO‐1, NRF2, and 4‐HNE. Moreover, CM increased uterine weight and serum superoxide dismutase in 17β‐estradiol‐treated OVX rats. These findings suggested that CM, particularly its component cordycepin, holds promise as a natural agent for mitigating weight gain, particularly in the context of postmenopausal obesity.

## 1. Introduction

Postmenopausal women face an increased risk of metabolic syndrome due to declining estrogen levels. In addition, menopausal syndrome, which is driven by autonomic imbalance, manifests through various symptoms, including vasomotor disorders, stiff shoulders, fatigue, and depression [1, 2]. Menopause significantly reduces the quality of life in many women. This diminishes their vitality in daily and social activities, resulting in substantial economic losses. Moreover, dyslipidemia, a metabolic syndrome, may increase the risk of cardiovascular diseases in women. Estrogen exerts multifaceted protective effects on lipid metabolism, including the inhibition of 3‐hydroxy‐3‐methylglutaryl coenzyme A (HMG‐CoA) reductase, the rate‐limiting enzyme in cholesterol synthesis, and the promotion of low‐density lipoprotein (LDL) clearance [3–5]. Indeed, the incidence of atherosclerotic diseases, such as ischemic heart disease and cerebrovascular accidents, shows a rising trend after menopause and eventually reaches levels comparable to those in men as age progresses [6]. Although hormone replacement therapy (HRT) can alleviate menopausal symptoms, concerns regarding increased risks of breast cancer and cardiovascular and thromboembolic events have led researchers and clinicians to seek safer, natural alternatives. Therefore, moderate consumption of natural compounds, known to reduce the risk of such diseases, may contribute to women’s health.

*Cordyceps militaris* (CM) belongs to the genus *Cordyceps*. Fungi of this genus parasitize the larvae of lepidopteran insects and subsequently grow to form fruiting bodies [7]. The dried fruiting bodies of *Cordyceps* have specific antifatigue effects without notable side effects and have long been used as a folk tonic in traditional Asian medicine [8–11]. Generally, CM contains cordycepin, ergosterol, and linoleic acid as the main bioactive compounds [11]. Cordycepin, in particular, has attracted considerable interest due to its potential anti‐inflammatory, antioxidant, and antitumor properties. In our previous study, the extract of CM derived from *Samia cynthia ricini* was found to inhibit androgen catabolism in an animal model of late‐onset hypogonadism (LOH) and to suppress testosterone‐induced prostate hypertrophy in an animal model of benign prostatic hyperplasia [12]. We previously found that cordycepin exhibited a growth‐inhibitory effect on cultured prostate cancer cells [13], suggesting that the in vivo observed effects may be attributed to cordycepin, although the details remain unclear. The extract derived from natural *Cordyceps sinensis* [14] and its key active compound cordycepin [15] have been reported to promote lipolysis via the activation of hormone‐sensitive lipase (HSL) and inhibit lipid accumulation by suppressing protein kinase B and activating AMP‐activated protein kinase (AMPK). Recently, we demonstrated that both CM and cordycepin facilitated the reduction in lipid droplets and the activation of HSL in cultured 3T3‐L1 cells, as well as in a type 2 diabetic obese mouse model [16]. Moreover, cordycepin suppressed the expression of oxidative stress markers and cellular senescence markers and reduced LDL/VLDL cholesterol levels. However, the effects of CM on postmenopausal obesity remain unclear.

We hypothesized that CM supplementation would attenuate ovariectomy‐induced weight gain, modulate adipose tissue metabolism, and reduce oxidative stress in a murine model of postmenopausal obesity. Therefore, the aim of this study was to investigate the effects of CM on visceral adipose tissue metabolism using an animal model.

## 2. Materials and Methods

### 2.1. Preparation of the Extract From CM Fruit Body

A microbial strain of CM obtained from the National Institute of Technology and Evaluation (NBRC 100741, Chiba, Japan) was cultured in a synthetic defined (SD) medium. The SD medium contained polypeptone (1 g), agar (1.5 g), yeast extract (1 g), and glucose (3 g) in 100 mL of H_2_O. An efficient method for inducing the growth of CM fruit bodies parasitizing *Samia cynthia ricini* (Ryoukyu‐kaso in Japanese) was established, as previously described [12].

### 2.2. Animals and Tissue Preparation

C57BL/6N mice (8‐week‐old females; Japan SLC, Shizuoka, Japan) or Wistar–Imamichi rats (25‐day‐old and 8‐week‐old females; Institute for Animal Reproduction, Ibaraki, Japan) were maintained in a temperature‐ and light‐controlled room (12 h light, 12 h dark cycle). All animal care and surgical procedures were approved by the Institutional Animal Care Committees of Tokyo University of Pharmacy and Life Sciences (approval number: P22‐67), in accordance with the institutional guidelines for the care of experimental animals, which were in accordance with internationally accepted principles (US guidelines/NIH publication). To explore the effect of CM on menopausal obesity, ovariectomized (OVX) mice were fed a diet containing CM (0.1%) for 52 days (protocol 1, Figures 1 and 2). In our previous study using a diabetic obese mouse model, oral administration for 3 weeks at the same dosage tended to reduce LDL/VLDL levels and the expression of C/EBPβ in visceral fat [16]. To investigate the effect of CM on the action of estrogen, OVX rats were orally administered CM (20 mg/day) via gavage with 17β‐estradiol (1 μg, s.c.) daily for 10 days (protocol 2, Figures 3(a) and 3(b)). This CM dosage and administration method has been shown to elevate serum testosterone levels after 12 days in a rat model of LOH [12]. Component analysis revealed that 1 g of CM contained 5 mg of cordycepin. The day after the final treatment, the animals were sacrificed by cervical dislocation, and blood, periovarian visceral adipose tissue, and the uterus were isolated. Collected tissue samples were frozen and stored in liquid nitrogen. Rats, not mice, were used to obtain sufficient granulosa cells and serum. Immature 25‐day‐old rats were subcutaneously injected with 50 units of equine chorionic gonadotropin (eCG; Serotropin, ASKA Animal Health Co. Ltd., Tokyo, Japan), which stimulates the development of multiple growing follicles to obtain ovarian granulosa cells. In the first ovulation–induction model, eCG, which possesses strong follicle‐stimulating hormone (FSH) activity and weak luteinizing hormone (LH) activity, was used to obtain granulosa cells from developing follicles one day prior to ovulation.

FIGURE 1Effects of CM on body weight and visceral adipose metabolic markers in OVX mice. OVX mice were fed a diet containing 0.1% CM for 52 days. The total body weight (a) and the total food intake (b) are presented as the mean ± SEM of three to five mice. ^##^ *p* < 0.01 vs. Ctrl. ^∗^ *p* < 0.05, ^∗∗^ *p* < 0.01 vs. OVX. (c) Changes in *Pparg*, *Cebpa*, *Cebpb, Fabp4, Adipoq*, and *Lep* mRNA levels in the adipose tissue. *Gapdh* served as an internal control for RNA integrity. Data from three to five animals are shown. (d) Adipose tissue lysates were subjected to immunoblotting to detect C/EBPα/β, PPARγ, pAKT, AKT, pHSL, and ERα. GAPDH served as the loading control. Representative blots (left panel) and densitometry analysis (*n* = 3–5) from the immunoblot analysis (right panel) are shown. (e) Changes in *Cyp11a1*, *Hsd3b*, *Cyp17a1, Hsd17b1 ,* and *Cyp19a1* mRNA levels in the adipose tissue. *Gapdh* served as an internal control for RNA integrity. Data from three to five animals are shown. *p* < 0.05 was considered to represent statistical significance. The figure on the left depicts a metabolic pathway illustrating the effects of OVX on each enzyme.

**Figure:**
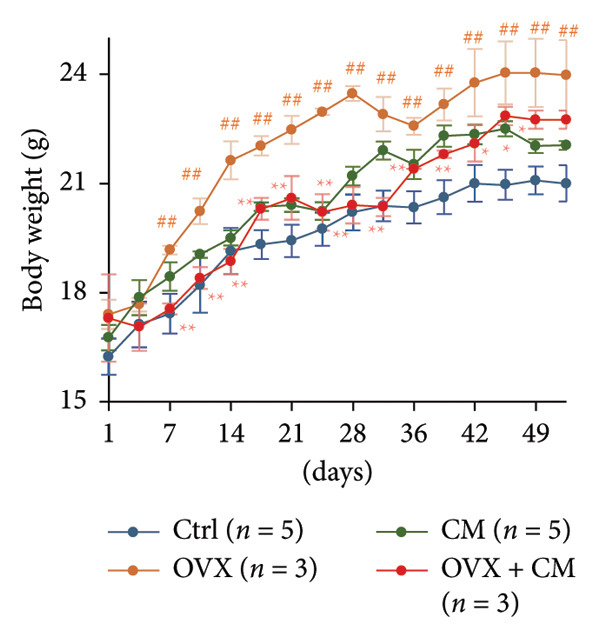
(a)

**Figure:**
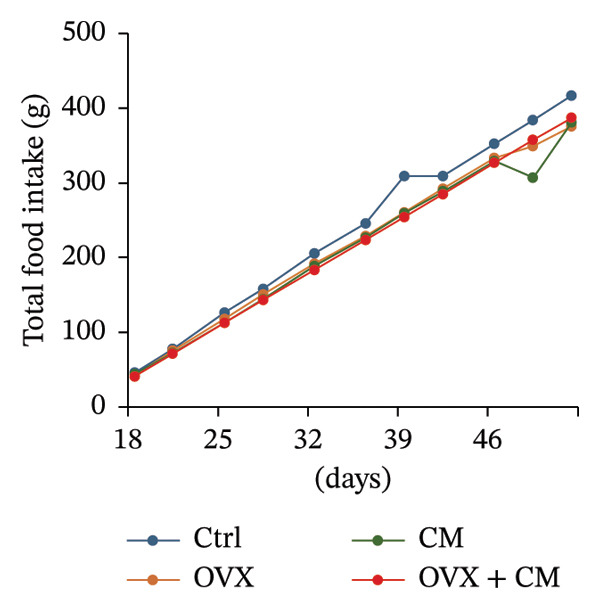
(b)

**Figure:**
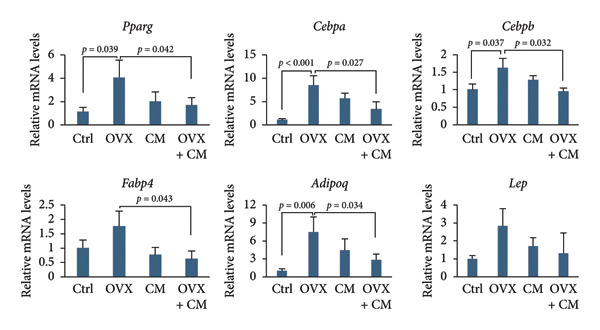
(c)

**Figure:**
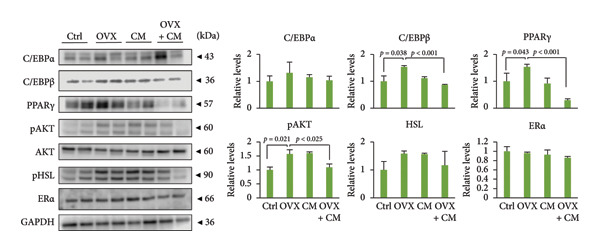
(d)

**Figure:**
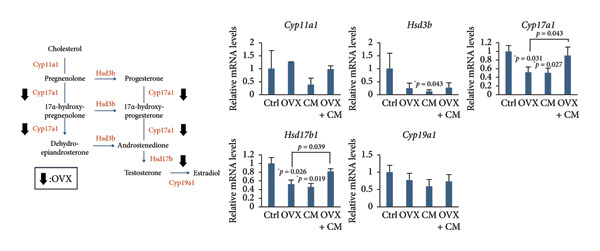
(e)

FIGURE 2Effects of CM on the senescence and cytoprotective markers in visceral adipose tissues in OVX mice. OVX mice were fed a diet containing 0.1% CM for 52 days. (a) Changes in *p21*, *p53*, and *Lmnb1* mRNA levels in the adipose tissue. *Gapdh* served as an internal control for RNA integrity. Data from three to five animals are shown. *p* < 0.05 was considered to represent statistical significance. (b) Adipose tissue lysates were subjected to immunoblotting to detect pNRF2, NRF2, KEAP1, HO‐1, and 4‐HNE. GAPDH served as the loading control. Representative blots (left panel) and densitometry analysis (*n* = 3–5) from the immunoblot analysis (right panel) are shown.

**Figure:**
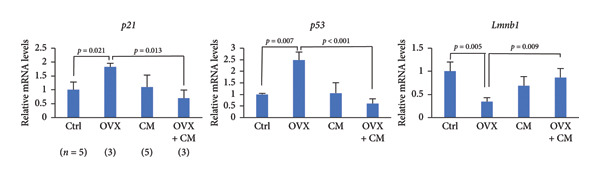
(a)

**Figure:**
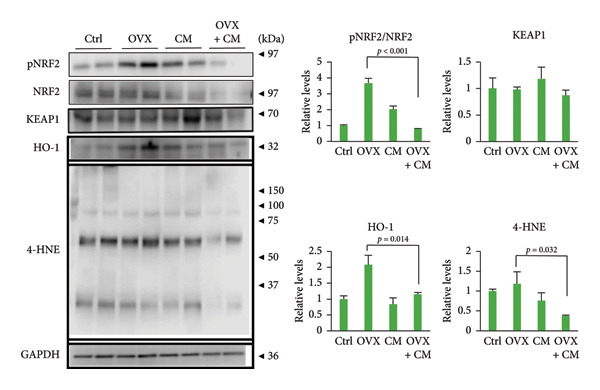
(b)

FIGURE 3Effects of CM on estradiol action and synthesis in rats. (a) Uterine weight on day 10 after administration of 17β‐estradiol (E2, 1 μg, s.c.) and/or CM (20 mg/rat, p.o.) to OVX rats. Data are presented as the mean ± SEM of five rats. *p* < 0.05 was considered to represent statistical significance. (b) The serum levels of SOD were measured by ELISA. Data from five animals are shown. (c) The serum levels of E2 were measured using ELISA. Data from five animals are shown. (d) The effect of CM on the secretion of E2 in primary rat ovarian granulosa cells. Granulosa cells were pretreated with CM (100 μg/mL) for 3 h and then stimulated with FSH (20 ng/mL) and LH (5 ng/mL) in the presence of CM for 18 h. The levels of E2 in the media were measured by ELISA. Data are represented as mean ± SEM from three independent experiments.

**Figure:**
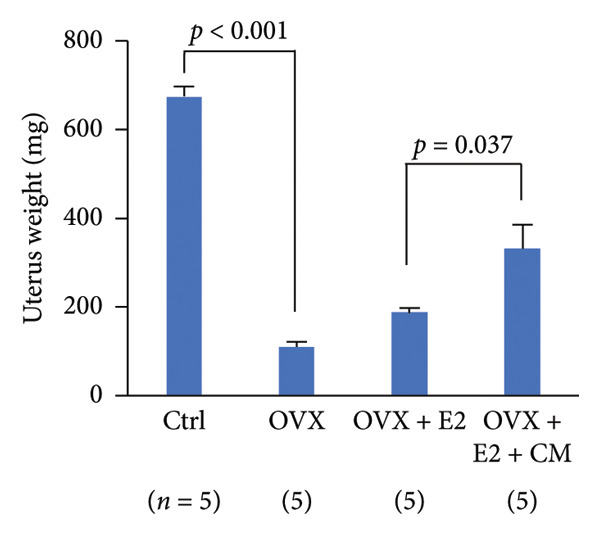
(a)

**Figure:**
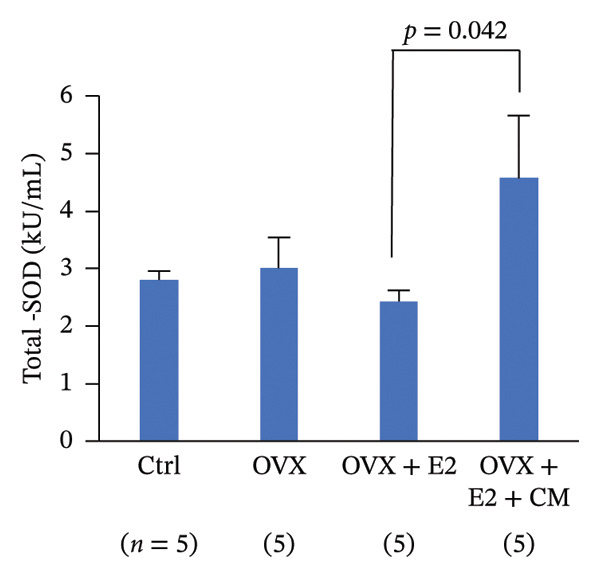
(b)

**Figure:**
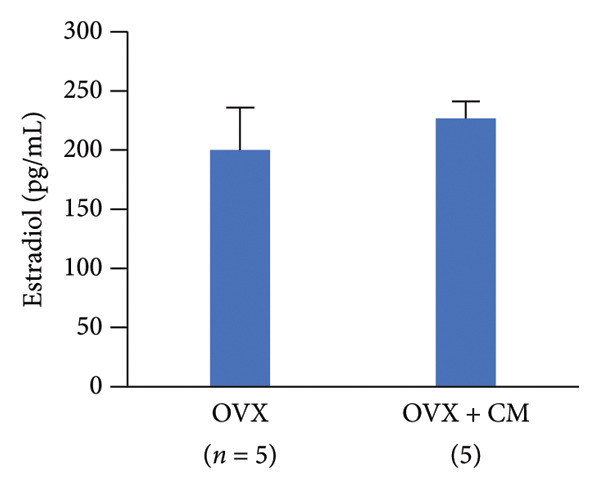
(c)

**Figure:**
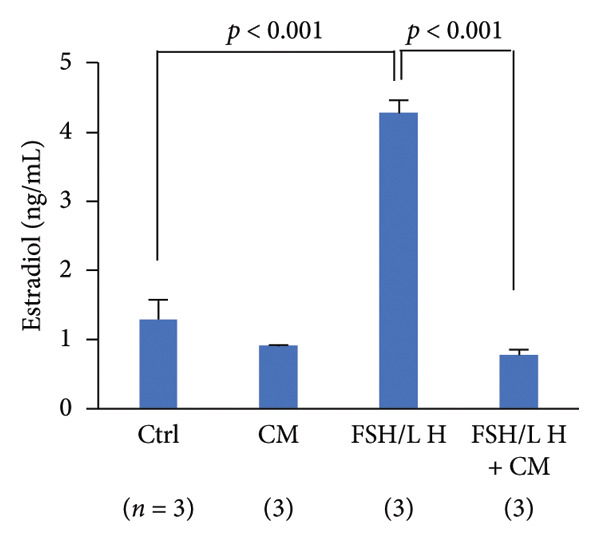
(d)

### 2.3. RNA Extraction and Quantitative RT‐PCR

Total RNA was extracted from frozen tissues using the ISOGEN reagent (Nippon Gene, Tokyo, Japan), according to the manufacturer’s protocol. Reverse transcription of the isolated RNA was performed using the LunaScript RT SuperMix Kit (New England Biolabs, Beverly, MA, USA), and the generated cDNA was subjected to qPCR amplification using PowerUP SYBR Green Master Mix (Thermo Fisher Scientific, Waltham, MA, USA). Primer sequences and amplicon sizes are listed in Table 1. Calibration curves were used to confirm that the amplification efficiencies of each target gene and the reference gene glyceraldehyde‐3‐phosphate dehydrogenase (*Gapdh*) were comparable. Sequence Detection System software v2.3 (Thermo Fisher Scientific) was used to determine the average threshold (Ct) values for each target [17].

**TABLE 1:** Primers for real‐time PCR.

Name accession no.	Sequence	Product length (bp)
*Gapdh*	F: 5′‐CAT​CAC​TGC​CAC​CCA​GAA​GAC​TG‐3′	153
NM_001289726.1	R: 5′‐ATG​CCA​GTG​AGC​TTC​CCG​TTC​AG‐3′	

*Pparg*	F: 5′‐GTA​CTG​TCG​GTT​TCA​GAA​GTG​CC‐3′	102
NM_001127330.2	R: 5′‐ATC​TCC​GCC​AAC​AGC​TTC​TCC​T‐3′	

*Cebpa*	F: 5′‐GCA​AAG​CCA​AGA​AGT​CGG​TGG​A‐3′	126
NM_007678.3	R: 5′‐CCT​TCT​GTT​GCG​TCT​CCA​CGT​T‐3′	

*Cebpb*	F: 5′‐CAA​CCT​GGA​GAC​GCA​GCA​CAA​G	113
NM_001287738.1	R: 5′‐GCT​TGA​ACA​AGT​TCC​GCA​GGG​T	

*Fabp4*	F: 5′‐TGA​AAT​CAC​CGC​AGA​CGA​CAG​G‐3′	125
NM_024406.4	R: 5′‐GCT​TGT​CAC​CAT​CTC​GTT​TTC​TC‐3′	

*Adipoq*	F: 5′‐AGA​TGG​CAC​TCC​TGG​AGA​GAA​G‐3′	156
NM_009605.5	R: 5′‐ACA​TAA​GCG​GCT​TCT​CCA​GGC​T‐3′	

*Lep*	F: 5′‐GCA​GTG​CCT​ATC​CAG​AAA​GTC​C‐3′	131
NM_008493.3	R: 5′‐GGA​ATG​AAG​TCC​AAG​CCA​GTG​AC‐3′	

*Cyp11a1*	F: 5′‐TGC​TCA​ACC​TGC​CTC​CAG​ACT​T‐3′	150
NM_019779.4	R: 5′‐ACT​GGC​TGA​AGT​CTC​GCT​TCT​G‐3′	

*Hsd3b*	F: 5′‐AGA​ACT​GCA​GGA​GGT​CAG​AGC​T‐3′	118
NM_008293.4	R: 5′‐GGC​ATC​CAG​AAT​GTC​TCC​TTC​C‐3′	

*Cyp19a1*	F: 5′‐CGA​AGC​AGC​AAT​CCT​GAA​GGA​G‐3′	134
NM_007810.4	R: 5′‐CCA​AGT​CCA​CAA​CAG​GCT​GGT​A‐3′	

*Cyp17a1*	F: 5′‐AGC​TCT​GTG​CTG​AAC​TGG​ATC​C‐3′	108
NM_007809.3	R: 5′‐AGA​CGG​TGT​TCG​ACT​GAA​GCC​T‐3′	

*Hsd17b1*	F: 5′‐TTC​TGC​CAG​ACA​TGA​AGA​GGC​G‐3′	110
NM_010475.2	R: 5′‐CGC​AAA​CTT​GCT​GGC​ACA​GTA​C‐3′	

*p21*	F: 5′‐TCG​CTG​TCT​TGC​ACT​CTG​GTG​T‐3′	124
NM_007669.5	R: 5′‐CCA​ATC​TGC​GCT​TGG​AGT​GAT​AG‐3′	

*p53*	F: 5′‐CAG​CCA​AGT​CTG​TTA​TGT​GCA​C‐3′	158
NM_001127233.1	R: 5′‐CGT​CAT​GTG​CTG​TGA​CTT​CTT​G‐3′	

*Lmnb1x*	F: 5′‐AGG​AAG​AGC​TGG​AGC​AGA​CCT​A‐3′	150
NM_010721.2	R: 5′‐GCA​GGT​TAG​AGA​GCT​GTG​AGG​A‐3′	

*Note:* F: forward; R: reverse.

### 2.4. Western Blotting

Adipose tissues were lysed with RIPA buffer (Thermo Fisher Scientific), according to the manufacturer’s instructions. The constituent proteins were separated using sodium dodecyl sulfate‐polyacrylamide gel electrophoresis (SDS‐PAGE) and transferred onto polyvinylidene difluoride membranes (Bio‐Rad Laboratories, Hercules, CA, USA) using Trans‐Blot Turbo (Bio‐Rad). After incubation for 2 h with the blocking buffer (Bullet Blocking One, Nacalai Tesque, Kyoto, Japan), the membranes were incubated with primary antibodies specific for pHSL (1:2,000; Proteintech, Tokyo, Japan), ERK (1:2,000; Cell Signaling Technology: CST, Beverly, MA, USA), pERK (1:2,000; CST), PPARγ (1:2,000; Abcam, Cambridge, UK), C/EBPα (1:2,000; CST), C/EBPβ (1:2,000; CST), AKT (1:2,000; CST), pAKT (1:2000; CST), estrogen receptor α (ERα, 1:2000; Merck Millipore, Burlingame, MA, USA), pNRF2 (1:2000; Selleck, Kanagawa, Japan), NRF2 (1:2,000; Proteintech), KEAP1 (1:2,000; Proteintech), HO‐1 (1:2,000; Proteintech), 4‐hydroxy‐2‐nonenal (4‐HNE; 1:2000, JaICA, Shizuoka, Japan), or GAPDH (1:5,000, Fujifilm Wako Pure Chemical Corp.). Immunoreactive bands were detected using an enhanced chemiluminescence kit (Merck Millipore) after incubation with horseradish peroxidase‐labeled goat antirabbit or antimouse IgG (1:5,000; Vector Laboratories, Burlingame, CA, USA). Signals were detected using a C‐DiGit Blot Scanner (LI‐COR, Lincoln, NE, USA), and the relative densities of the bands were assessed through densitometric analysis of the digitalized autographic images using Image Studio DiGit software (v5.2) [17].

### 2.5. Isolation of Ovarian Granulosa Cells

Granulosa cells from eCG‐primed rats were harvested as previously described [18, 19]. Ovaries collected 48 h after eCG injection were placed in Hanks’ balanced salt solution and granulosa cells were collected by puncturing mature preovulatory follicles using a 27 G needle under a dissecting microscope. The cells were centrifuged at 800 × *g*, washed twice in PBS, and seeded at 2 × 10^5^ cells/well onto a 24‐well tissue culture dish coated with collagen type I. Cells were initially cultured in Dulbecco’s Modified Eagle Medium (DMEM, FUJIFILM Wako Pure Chemical Corp., Osaka, Japan) supplemented with 10% fetal bovine serum (FBS) until reaching subconfluency. Subsequently, the cells were cultured in a chemically defined, serum‐free DMEM supplemented with 5 μg/mL transferrin, 40 ng/mL hydrocortisone, 4 mg/mL BSA, 30 ng/mL androstenedione, 1 mg/mL fibronectin, 4 μg/mL insulin, 100 ng/mL FSH (NIDK ovine FSH‐19‐SIAFP), and 10 ng/mL LH (NISDK ovine LH26‐AFP‐551B).

### 2.6. Measurement of Estradiol

The concentrations of 17β‐estradiol (E2) in the serum and in culture media from granulosa cells were determined through enzyme‐linked immunosorbent assay (ELISA; Estradiol ELISA Kit, Cayman Chemical, Ann Arbor, MI, USA), according to the manufacturers’ instructions.

### 2.7. Superoxide Dismutase (SOD) Activity Assay

Serum SOD activity was determined using the SOD assay kit‐WST (Dojindo), according to the manufacturer’s instructions [20]. This assay is a colorimetric method for evaluating SOD activity by measuring the amount of water‐soluble formazan produced from the tetrazolium salt WST‐1, with absorbance quantified at 450 nm.

### 2.8. Statistical Analysis

Data are expressed as mean ± SEM and were compared using Dunnett’s test in R software (v4.0.5). For comparisons involving more than two groups, analysis of variance (ANOVA) was conducted, followed by Tukey’s post hoc test for multiple comparisons. Differences were considered statistically significant at *p* < 0.05.

## 3. Results

### 3.1. Effects of CM on Body Weight and Visceral Adipose Tissue in OVX Mice

To investigate whether CM affects obesity induced by estrogen reduction, OVX mice were fed a diet containing 0.1% CM for 52 days. OVX significantly increased body weight (*p* = 0.009), and CM suppressed OVX‐induced weight gain (Figure 1(a)). Notably, CM‐treated non‐OVX animals exhibited a trend toward increased body weight compared with the intact control group (*p* = 0.038 on day 52). Daily food intake was not altered by OVX or CM treatment (Figure 1(b)). In visceral adipose tissue, the expression of adipogenic markers (*Pparg*: 2.7‐fold, *Cebpa*: 8.6‐fold, *Cebpb*: 1.6‐fold, *Fabp4*: 1.7‐fold, and *Adipoq*: 8.3‐fold vs. control) was elevated by OVX and significantly suppressed by CM intake (Figure 1(c)). Consistent with mRNA expression, CM treatment suppressed the OVX‐induced elevations in the protein levels of C/EBPβ, PPARγ, and pAKT (Figure 1(d)). However, CM did not affect the ERα or adipolysis marker pHSL. To further investigate the impact of CM on E2 metabolism in adipose tissue, the expression levels of steroid hormone biosynthesis enzymes were assessed. OVX significantly decreased *Cyp17a1* (50% vs. control) and *Hsd17b1* (53%) levels, which were ameliorated by CM intake (Figure 1(e)). CM did not affect *Cyp11a1*, *Hsd3b*, or *Cyp19a1* levels.

### 3.2. Effects of CM on the Senescence and Cytoprotective Markers in Visceral Adipose Tissues in OVX Mice

Oxidative stress and cellular senescence levels are reported to be elevated in the visceral adipose tissue of obesity [21, 22]. These levels are pivotal contributors to the development of age‐related and chronic inflammatory diseases, underscoring the importance of their assessment. These parameters serve as crucial indicators to determine the effectiveness of antioxidant‐ and senescence‐targeting interventions. Therefore, OVX mice were examined for the effects of CM on cellular senescence and oxidative stress in adipose tissues. OVX upregulated the expression of senescence markers *p21* (7.9‐fold vs. control) and *p53* (2.1‐fold), but suppressed that of *Lmnb1* (0.3‐fold) encoding nuclear lamin B1; however, these OVX‐induced changes were reversed by CM treatment (Figure 2(a)). Levels of the cytoprotective factor HO‐1 were increased in OVX mice and were significantly suppressed by CM (Figure 2(b)). Moreover, CM treatment significantly reduced the levels of 4‐HNE, an indicator of lipid peroxidation, and NRF2 phosphorylation, which are commonly used markers associated with oxidative stress, in OVX mice (Figure 2(b)).

### 3.3. Effects of CM on Estradiol Action and Synthesis in Rats

Given that the rat endometrium generally exhibits higher responsiveness and proliferative effects to estrogen compared to that of mice [23], we next investigated the modulatory effects of CM on estrogen action in the uterus of OVX rats, as well as its effects on estrogen production by ovarian granulosa cells. OVX rats were orally administered CM (20 mg/day) daily for 10 days. OVX‐induced reduction in uterine weight (86% reduction vs. control) was elevated after E2 treatment, and these levels were significantly enhanced by CM (175% vs. control, Figure 3(a)). CM increased the levels of serum SOD (188% vs. E2 alone), a catabolic enzyme of reactive oxygen species (ROS; Figure 3(b)), but CM did not alter serum E2 levels (Figure 3(c)). Additionally, we examined the effects of CM on E2 production in cultured ovarian granulosa cells. FSH and LH increased E2 levels (3.45‐fold vs. control) in the culture medium, which were clearly reduced by CM treatment (18.4% vs. FSH/LH, Figure 3(d)).

## 4. Discussion

**FIGURE 4:**
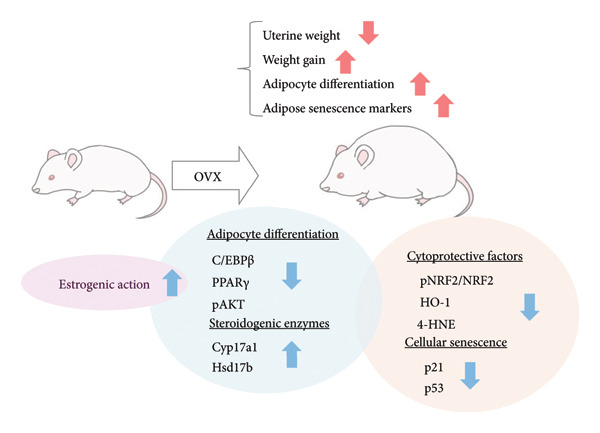
Diagram illustrating the effect of CM on adipose tissue changes associated with obesity in OVX animals. OVX: ovariectomy; red arrow: changes in OVX; blue arrow: effects of CM on changes after OVX.

This study demonstrated that CM reduced the body weight in OVX‐induced menopause model mice. CM decreased both mRNA and protein levels of the adipogenic markers PPARγ and C/EBPα/β in adipose tissue. The upregulation of these transcription factors, which are critical for the differentiation of preadipocytes into mature adipocytes, has been associated with enhanced adipocyte differentiation and lipid accumulation within the visceral adipose tissue. The elevated expression of these genes in the OVX mice could be interpreted as a physiological consequence of estrogen deficiency. Our previous study also demonstrated that CM and its main component, cordycepin, inhibited adipocyte differentiation of 3T3‐L1 cells, accompanied by downregulation of C/EBPβ and PPARγ. CM may inhibit preadipocyte differentiation into adipocytes by suppressing the expression of these transcription factors. Interestingly, an extract of mulberry leaves fermented with *Cordyceps militaris* has also been shown to inhibit lipogenesis and promote lipolysis in high‐fat diet‐induced obesity [22, 24]. In our previous study, CM and cordycepin reduced circulating LDL/VLDL levels and decreased C/EBPβ expression in a diabetic obese mouse model [16]. Thus, CM derived from *Samia cynthia ricini* might help alleviate lipid metabolism dysregulation in postmenopausal women. The present study suggests that the antiobesity effects of CM may be mediated, at least in part, through the suppression of cellular senescence and cytoprotective reaction in adipose tissue, accompanied by downregulation of the adipogenic transcription factors C/EBPβ and PPARγ, as well as restoration of the expression of local steroid‐metabolizing enzymes (Figure 4). Postmenopausal women are known to have an increased risk of metabolic syndrome and cardiovascular disease due to visceral fat accumulation. The present findings suggest that CM may serve as a natural candidate for functional food or pharmaceutical development to target obesity in postmenopausal women.

Weight gain has also been reported in menopause model rats [25]. In these animals, estrogen replacement has been observed to induce anorectic effects under a high‐fat diet, implicating ghrelin suppression as a potential mechanism. Estrogen plays a critical role in regulating feeding behavior and body weight in female mammals. Previous studies using rat models have consistently reported increased food intake and body weight after OVX. However, in the present study, no significant changes in food intake were observed in OVX mice. This discrepancy may be attributed to multiple factors. First, there may be species‐specific differences in the feeding behavior between rats and mice, suggesting that the effects of OVX are less pronounced in mice. Additionally, the mice used in this study were relatively young (8 week old), and their hormonal sensitivities and metabolic responses may differ with increasing age. Furthermore, adrenal‐derived estrogen might be secreted in a compensatory manner following OVX, and such endogenous hormonal mechanisms may attenuate the impact on appetite‐regulating centers in these mice. Environmental conditions also represent critical factors influencing feeding behavior. For example, behavioral differences arise between animals housed individually [25] versus in groups, with varying degrees of stress. In the present study, group housing was implemented, which may have masked the effects of OVX. Moreover, because a standard chow diet was used instead of a high‐fat diet, the modulatory effects of estrogen on dietary preferences may have been less evident. Taken together, the absence of increased food intake following OVX in this study may be attributed to a complex interplay of factors, including species differences, age, hormonal milieu, housing conditions, and dietary composition.

Previous studies have suggested that cordycepin, a key component of CM, may suppress adipogenesis by activating AMPK and inhibiting mechanistic target of rapamycin complex 1 (mTORC1) activation mediated by AKT [13]. Within the PI3K/AKT/mTOR signaling axis, AKT1 deficiency in mice has been shown to promote energy expenditure and prevent diet‐induced obesity [26]. Furthermore, inhibition of PI3Kα‐AKT1 has been reported to increase energy expenditure in adipose tissue, reduce body weight gain, and improve insulin resistance in diet‐induced obese mice [27]. AKT1 is also involved in regulating immune inflammation. In macrophages stimulated with LPS, activation of AKT1 promotes NFκB signaling and upregulates inflammatory mediators [28, 29]. These findings are consistent with our current observation that CM suppressed the OVX‐induced increase in Akt expression, likely mediated by cordycepin. Additionally, in a benign prostatic hyperplasia model, cordycepin was reported to enhance the phosphorylation of AMPK, while inhibiting that of AKT (p‐AKT) [30], suggesting that a similar signaling mechanism may operate in adipocytes. Cordycepin has been shown to increase ERK1/2 phosphorylation [31]. ERK1/2 regulates HSL phosphorylation, suggesting a potential indirect role in lipid metabolism. Cordycepin is a natural adenosine analog that binds to adenosine receptors [32]. It does not stimulate the adenosine P1A1, P1A2a, or P1A2b receptors during preadipocyte differentiation. Instead, cordycepin acts on preadipocytes via adenosine transporters [15]. In the OVX mouse model used in this study, CM containing cordycepin may have affected preadipocytes via adenosine transporters, thereby inhibiting adipogenesis. Although cordycepin has also been suggested to exert antiproliferative effects through adenosine A3 receptors [33], the involvement of this pathway in adipocyte differentiation remains to be elucidated. Recently, a study employing an integrative strategy combining network pharmacology, quantitative transcriptomic analysis, and molecular docking systematically identified therapeutic targets for diet‐induced obesity [34]. Multiple potential targets and pathways related to the antiobesity effects of cordycepin, including AKT1 and GSK3B, have been identified. These targets are involved in key signaling pathways such as insulin signaling, HIF‐1, FoxO, and lipid metabolism/atherosclerosis. Thus, the antiadipogenic effects of cordycepin are likely not mediated by a single pathway but rather through a complex interplay of multiple signaling networks that collectively suppress adipocyte differentiation.

Furthermore, CM increased the uterine weight induced by E2 administration in OVX animals, suggesting that CM exerts estrogen‐like activity. However, given that serum E2 levels remained unchanged, it is possible that CM activates ERs locally or promotes uterine proliferation through alternative growth factors, such as insulin‐like growth factor (IGF)‐1. Although no changes in ERα expression were observed in adipose tissue, it has been reported that *Cordyceps* extract increases ERα phosphorylation levels in human breast cancer cells [17]. These findings suggest that CM may enhance ERα‐mediated activity in the uterus independently of E2 synthesis, supporting our results. Interestingly, previous reports using OVX rats suggest that other CM may contribute to antiobesity effects in menopause by exhibiting ERα agonistic activity [35]. Consistent with the previously mentioned pharmacological properties of cordycepin, this compound is also predicted to interact with multiple obesity‐ and menopause‐related signaling pathways, including the insulin, MAPK, PI3K‐AKT, and estrogen pathways. In cultured granulosa cells, CM markedly suppressed gonadotropin‐stimulated E2 production. Although the mechanism remains unclear, CM might downregulate the transcription or translation of key steroidogenic enzymes such as CYP19A1, StAR, and CYP11A1. Figure 1 shows that CM alone suppressed the expression of *Hsd3b*, *Cyp17a1*, and *Hsd17b1*, genes involved in sex hormone synthesis, in adipose tissue. Another possible mechanism involves the interference with gonadotropin receptor signaling. Gonadotropins enhance aromatase expression via the cAMP/PKA pathway. CM may attenuate this pathway by downregulating FSH receptor or LH receptor expression or by suppressing cAMP production. Furthermore, CM might modulate granulosa cell differentiation or induce mild apoptosis, thereby reducing steroidogenic capacity. We examined the mRNA expression levels of steroidogenic and steroid‐metabolizing enzymes in the periovarian visceral adipose tissue of OVX mice. In the steroid metabolic pathway, the expression of *Cyp17a1*, which is involved in the metabolism of pregnenolone to dehydroepiandrosterone (DHEA), and *Hsd17b1*, which converts androstenedione to testosterone and estrone to E2, was decreased in OVX mice and improved with CM treatment. However, the expression levels of *Hsd3b* and *Cyp19a1* did not change. These results suggested that CM may increase local E2 levels in adipose tissue by attenuating E2 metabolism. Enhanced insulin sensitivity, partly due to increased E2 action, may contribute to these antiobesity effects.

This study also showed that CM reduced the levels of cellular senescence and cytoprotective markers in the adipose tissues of OVX mice. Furthermore, elevated serum SOD activity was also detected after CM treatment in OVX animals. Adipose tissue is a major source of ROS production [36–38]. Both mature adipocytes and preadipocytes are highly sensitive to redox changes. Hypertrophic and dysfunctional adipocytes increase ROS production, enhancing oxidative stress in the adipose tissue microenvironment through oxidative damage of accumulated lipids, leading to lipid peroxidation and impaired preadipocyte differentiation [39]. Cells with elevated ROS production activate antioxidant signals to defend against this, including the activation of the NRF2‐HO‐1 pathway. In OVX mice, an increase in adipose HO‐1 levels was observed, which was suppressed by CM treatment. Since SOD expression is also regulated by pathways other than Nrf2, CM may have enhanced SOD activity through Nrf2‐independent pathways. Overall, these changes suggest multifaceted antioxidant effects of CM. Senescence plays a significant role in the onset and progression of several chronic diseases, including obesity [40, 41]. Senescent phenotypes can also arise due to oxidative stress [42]. In this study, administration of CM to ovariectomized mice resulted in reduced lipid peroxidation in adipose tissue, accompanied by decreased NRF2 phosphorylation and HO‐1 expression. This reduction in oxidative stress likely diminishes the need for stress‐induced cellular defense responses involving NRF2 and HO‐1. Previous studies using diabetic obese mouse models have demonstrated increased oxidative stress and cellular senescence markers in adipose tissue [16]. Cordycepin prevents radiation‐induced cellular senescence via the NRF2 and AMPK pathways [43]. These findings suggest that CM containing cordycepin may improve menopausal obesity by modulating cytoprotective signaling and attenuating cellular senescence in adipose tissue, while also enhancing serum SOD activity and reducing oxidative stress markers. Thus, CM appears to exert a preventive antioxidant effect by limiting intracellular oxidative stress load. Consequently, senescence markers and DNA damage‐related factors such as *p21* and *p53* were downregulated, implying that CM mitigates cellular senescence and inflammation. Taken together, these observations support the hypothesis that CM may function as a preventive antioxidant, helping to preserve a low‐stress cellular milieu.

The present findings are based on animal experiments using OVX mice and rats, as well as in vitro cell‐based studies. However, these results may not be directly translatable to humans. Animal models do not fully replicate human pathophysiology, such as postmenopausal obesity, and inherent interspecies differences exist in pharmacokinetics, including ADME, pharmacodynamics, and safety profiles. Thus, the application of CM in clinical practice requires further research involving human participants, including well‐designed clinical trials. Our findings suggested that CM and cordycepin suppress the expression of transcription factors involved in adipocyte differentiation (e.g., C/EBPβ and PPARγ) and mitigate oxidative stress and cellular senescence in adipose tissue. However, how these mechanisms contribute to the overall antiadipogenic effects of CM and how they interact remain unclear. Additionally, other components of CM may also play a role. Notably, the CM used in this study was derived from a specific strain that parasitizes *Samia cynthia ricini*. Its chemical composition, including active constituents, such as cordycepin, may vary depending on the strain and cultivation method used. Therefore, it remains unclear whether the observed antiobesity effects and mechanisms apply to extracts from other sources or under different cultivation conditions.

This study focused primarily on visceral adipose tissue. However, adipose tissue consists of both visceral and subcutaneous fat depots, which differ in their physiological roles and metabolic characteristics. The potential effects of CM on subcutaneous adipose tissue remain unclear. Moreover, it is difficult to capture a full systemic picture, including the impact on other organs, whole‐body energy metabolism, and hormonal balance, based on this study alone. Further investigations of these aspects are warranted. Additionally, the duration of the animal experiments was relatively short (52 days). Assessing the long‐term efficacy and potential risks of chronic intake of CM, including safety and possible adverse effects, requires extended observation periods. Although the present findings suggest that CM may be effective against postmenopausal obesity, particularly visceral fat accumulation, more comprehensive studies are necessary. Further elucidation of the underlying mechanisms and translational research, including clinical studies in humans, are essential to advance its potential therapeutic application.

In conclusion, CM inhibited the expression of key factors involved in adipocyte differentiation and maturation, while simultaneously suppressing OVX‐induced weight gain. Furthermore, CM restored the reduced expression of steroid metabolic enzymes in adipose tissue caused by OVX. CM also increased serum SOD activity and suppressed the expression of cytoprotective markers in adipose tissue, indicating its protective effects against oxidative stress‐induced damage. Furthermore, CM increased uterine weight under E2 stimulation, which suggests the potential enhancement of ER signaling or inhibition of E2 catabolism. These findings indicated that CM could be a potential natural material for improving obesity and lipid metabolic disorders associated with estrogen reduction during menopause.

## Author Contributions

Kazuya Kusama and Kazuhiro Tamura conceived and designed the experiments; Kazuya Kusama, Chisaki Fujii, Kanoko Yoshida, and Mikihiro Yoshie performed the experiments and analyzed the data; Kazuya Kusama and Kazuhiro Tamura wrote the manuscript. Kazuya Kusama, Chisaki Fujii, Kanoko Yoshida, Mikihiro Yoshie, Yoshiyuki Adachi, Hiroaki Miyaoka, and Kazuhiro Tamura coordinated the project and contributed to data analysis and interpretation of the results.

## Funding

This study was funded by the Strategic Foundational Technology Improvement Support Operation​ (2022).

## Disclosure

All the authors have read and agreed to the published version of the manuscript.

## Conflicts of Interest

The authors declare no conflicts of interest.

## Data Availability

The data that support the findings of this study are available from the corresponding author upon reasonable request.
